# Clonal MDS/AML cells with enhanced TWIST1 expression reprogram the differentiation of bone marrow MSCs

**DOI:** 10.1016/j.redox.2023.102900

**Published:** 2023-09-21

**Authors:** Hongjiao Li, Yi Wang, Fenfang Yang, Shuang Feng, Kaijing Chang, Xinwen Yu, Feng Guan, Xiang Li

**Affiliations:** a Key Laboratory of Resource Biology and Biotechnology of Western China, Ministry of Education, Provincial Key Laboratory of Biotechnology, College of Life Sciences, Northwest University, Xi'an, China; b Department of Hematology, Provincial People's Hospital, Xi'an, China; c Institute of Hematology, School of Medicine, Northwest University, Xi'an, China

**Keywords:** MDS/AML, BMMSCs, Osteogenic/adipogenic differentiation, TWIST1, ROS

## Abstract

Bone marrow-derived mesenchymal stem cells (BMMSCs) derived from myelodysplastic syndrome (MDS) and acute myeloid leukemia (AML) patients often show a shift in the balance between osteoblastogenesis and adipogenesis. It was suggested that BMMSCs can potentially undergo reprogramming or educational processes. However, the results of reprogrammed differentiation have been inconclusive. In this study, clinical samples, co-culture models and mouse models were employed to explore the association of MDS/AML clonal cells and BMMSCs differentiation. We found that clonal MDS/AML cells promoted adipogenic differentiation and inhibited osteogenic differentiation of BMMSCs, which in turn promoted MDS expansion. Mass spectrometry and cytokine array were used to identify the molecules to drive the BMMSCs differentiation in MDS/AML. Mechanistically, highly expressed transcription factor TWIST1 in clonal MDS/AML cells induces MDS/AML cells to secrete more IFN-γ, which can induce oxidative stress through STAT1-dependent manner, ultimately causing enhanced adipogenic differentiation and inhibited osteogenic differentiation in BMMSCs. Overall, our findings suggest that targeting the driving oncogenes in malignant clonal cells, such as TWIST1, may offer new therapeutic strategies by remodeling the surrounding bone marrow microenvironment in the treatment of MDS/AML and other hematopoietic malignancies.

## 1 Introduction

Myelodysplastic syndrome (MDS) is myeloid neoplasms characterized by clonal proliferation of hematopoietic stem cells, recurrent genetic abnormalities, ineffective hematopoiesis, peripheral-blood cytopenia, and a high risk of evolution to acute myeloid leukemia (AML) [1]. Recent evidence has revealed that the bone marrow microenvironment (BMME), including mesenchymal stem cells and hematopoietic stem cell niche cells, is another key contributor to disease initiation and progression [2]. Malignant clonal cells can modify the BMME via aberrant production of secreted factors, and the resulting dysfunctional BMME further promotes clonal expansion [3,4]. It suggested the importance of understanding the complex interactions between malignant clonal cells and the BMME in the development of myeloid neoplasms.

Bone marrow mesenchymal stem cells (BMMSCs), which are part of the BMME, have the ability to differentiate into various types of cells, including osteoblasts, adipocytes, and chondroblasts [[4], [5], [6]]. These cells are known to play an important role in regulating hematopoiesis under physiological conditions. Several reports have documented the differential abnormality of BMMSCs in human myeloid malignancies [[7], [8], [9]]. For instance, a cohort study involving 106 samples from patients with MDS observed a reduction in osteogenic differentiation potential in BMMSCs [7,10]. Another group suggested that BMMSCs from patients with MDS/AML exhibit diminished osteogenic differentiation and enhanced adipogenic differentiation [[11], [12], [13]]. Additionally, the levels of two key factors for osteogenic differentiation, Osterix and RunX2, were found to be reduced in BMMSCs, and the number of osteoblasts was significantly decreased in patients with low-risk MDS, indicating impaired osteogenic differentiation of MDS-derived BMMSCs [14,15]. AML cell-derived exosomes can induce MSCs toward an adipogenic differentiation accompanied by a metabolic switch from glycolysis to oxidative phosphorylation-dependent manner [16]. In turn, the altered differentiation potential of BMMSCs can generate a protumoral microenvironment for clonal cell growth [7,11,14,17]. For example, leukemic cells can reprogram bone marrow (BM) adipocytes to support the survival and proliferation of malignant cells from patients with AML [17]. Although these findings evidence that deficiencies in adipogenic and osteogenic differentiation do exist among BMMSCs in these patients, the molecular mechanism of such differentiation defects with clonal MDS/AML cells remains poorly understood.

In our current study, we observed a reduction in osteogenic differentiation and an increase in adipogenic differentiation of BMMSCs from patients with MDS/AML. Additionally, we discovered that murine model following injection of BM from patients with MDS/AML can cause a shift in adipogenesis over osteoblastogenesis in mice bone marrow. We found the differentiation defect was associated with enhanced expression of transcription factor TWIST1 in MDS/AML clonal cells. TWIST1 is previously showed to be dysregulated in MDS/AML and implicated in the effectiveness of decitabine therapy [18,19]. Subsequently, we investigated the mechanisms how increased TWIST1 modulate the interaction between BMMSC differentiation and clonal cell growth.

## 2 Materials and methods

### 2.1 Isolation and culture of primary BMMSCs

BMMSCs were isolated from healthy donors (HD) or MDS/AML patients (Table 1) as described previously [20]. Briefly, mononuclear cells were separated from the BM with an equal volume of Ficoll solution (Solarbio, Beijing, China) and cultured in MSC basal medium (MSCBM, Dakewe Biotech, Beijing, China) containing 5% serum replacement (UltraGROTM-Advanced, Helios, USA) and 1% penicillin/streptomycin (Gibco, Grand Island, NY, USA) at 37 °C in a 5% CO_2_ atmosphere.

**Table 1:** Characteristics of patients with MDS/AML and healthy subjects.

Diagnosis	Age (yr)	Gender	Cytogenetics	BM Cellularity	Marrow Blast Count
AML	59	M	normal	normal	33%
AML	60	M	normal	hyper	58%
MDS	58	M	+8	normal	6.5%
AML	69	M	normal	hyper	41%
AML	50	F	t(8; 21)	hyper	63.5%
AML	80	M	t(8; 21)	hyper	36%
MDS	54	M	normal	normal	16.8%
AML	73	F	normal	hyper	43%
AML	48	M	t(12; 22)	hyper	78%
AML	48	M	normal	hyper	45%
AML	64	M	normal	hyper	91%
AML	31	F	normal	hyper	53%
AML	48	F	normal	hyper	61%
MDS	35	M	normal	hyper	12%
MDS	31	M	normal	hyper	14%
AML	78	F	normal	normal	40%
MDS	65	M	normal	normal	6.5%
MDS	62	M	normal	normal	8%
AML	48	M	t [8,21]	hyper	23.5%
AML	58	M	normal	hyper	29%
MDS	66	F	+8	normal	23%
AML	43	F	t [8,21]，+8	hyper	29%
AML	58	F	normal	normal	32%
AML	32	F	t [8,21]	hyper	36%

AML	49	M	t [8,21]	hyper	30.6%
MDS	66	M	+8	normal	2.5%
MDS	62	M	normal	hyper	13%
HD	22	F	–	–	–
HD	22	F	–	–	–
HD	23	M	–	–	–
HD	25	F	–	–	–
HD	67	F	–	–	–
HD	48	M	–	–	–
HD	69	M	–	–	–
HD	56	M	–	–	–
HD	43	F	–	–	–
HD	71	F	–		–
HD	66	F	–		–
HD	75	M	–		–
HD	49	F	–	–	–
HD	58	M	–	–	–

CD34^+^ and CD45^+^ cells were sorted from mononuclear cells using a CD34 or CD45 microbeads Kit (Miltenyi Biotechnology company; Bergisch Gladbach, Germany) [21]. In accordance with the Declaration of Helsinki, written informed consent was obtained from all patients and healthy donors (HD). All protocols were reviewed and approved by the Research Ethics Committee of Northwest University.

Myeloid leukemia cell line KG1a was kindly donated by Prof. H. Joachim Deeg (Fred Hutchinson Cancer Center; Seattle, WA, USA). SKM1, a cell line established from MDS, was maintained and propagated in our lab as previously described [22,23]. These cells were all cultured as described previously [24].

### 2.2 Assessment of osteogenic differentiation

To induce osteogenic differentiation, BMMSCs were cultured in osteogenic differentiation medium containing β-glycerophosphate, glutamine, ascorbate, and dexamethasone (Cyagen, Suzhou, China) for 21 days. Afterward, the cells were stained with alizarin red solution (Cyagen), and the mineralized matrix was observed under an inverted microscope (ICX41, Sunny Optical Technology, Ningbo, China).

### 2.3 Assessment of adipogenic differentiation

To induce adipogenic differentiation, BMMSCs were cultured in adipogenic differentiation medium A (containing basal medium A, 1% penicillin-streptomycin, 10% FBS, insulin, glutamine, IBMX, rosiglitazone, and dexamethasone) and medium B (containing basal medium B, 1% penicillin-streptomycin, 10% FBS, glutamine, and insulin) (Cyagen) for 15 days, following the manufacturer's instructions. Adipogenesis was assessed by oil red O staining and visualized under an inverted microscope (ICX41).

### 2.4 Animal study

To establish patient-derived xenografts (PDXs), 6- to 8-week-old B-NSG™ mice (NOD-Prkdc^scid^*IL2rg*^tm1^/Bcgen, NSG; Biocytogen Pharmaceuticals, Beijing, China) were irradiated with 180 cGy. A total of 2 × 10^6^ mononuclear cells from the bone marrow of HD or MDS/AML patients (Table 1) were injected into NSG mice via the tail vein, as previously described [25]. Peripheral blood (100 μL) was collected from tail weekly after injection, and a total of 2 × 10^5^ mononuclear cells were analyzed by flow cytometry (FACS) with an antibody against human CD45 (BD Biosciences; Franklin Lakes, NJ, USA) using the ACEA Biosciences platform (San Diego, CA, USA). After 8 weeks, the mice were euthanized, and femur bones were collected to assess bone repair and osteoporosis.

For the xenotransplant assay, 6- to 8-week-old C57BL/6 mice (Biocytogen Pharmaceuticals) were irradiated with 3 Gy. KG1a cells or TWIST1-overexpressing KG1a (KG1a-TWIST1) cells (5 × 10^6^) were intrafemorally injected into the mouse BM within 12 h after irradiation. Mice were treated with IFN-γ (2 g/kg) (R&D Systems, Minneapolis, MN, USA) or Fludarabine (1 g/kg) (MedChemExpresss, Monmouth Junction, USA) 3 times per week. Peripheral blood was collected at 1 and 3 weeks after injection, and mononuclear cells were stained with anti-CD45 Ab and analyzed by FACS. After 3 weeks of injection, the mice were euthanized, and femur bones were collected.

### 2.5 Assessment of bone structure by micro-CT

After the mice were euthanized, their femur bones were extracted and fixed in 4% fresh paraformaldehyde for 48 h. The femur bones were scanned using a micro-CT scanner (NEMO micro-CT scan, NMC-100, PINGSENG HealthCare Inc., Shanghai, China) at a resolution of 16 μm, and the shin bone was scanned at a resolution of 10 μm. The resulting data were used to reconstruct a three-dimensional image of the femur using Avatar software.

### 2.6 Cell proliferation assay

Cells were stained with EdU Alexa Fluor 647 kit (Keygen; Jiangsu, China) according to the manufacturer's protocol. The stained cells were analyzed by FACS (ACEA Biosciences).

### 2.7 Mass spectrometry analysis

Proteins (100 μg) were denatured with 8 M urea, 10 mM DTT, and 20 mM IAM (Sigma‐Aldrich), and then digested with two proteases: lysyl endopeptidase (Wako Pure Chemical; Osaka, Japan) and trypsin (Promega; Madison, WI, USA). The resulting peptides were collected, purified using Oasis HLB cartridges (Waters; Milford, MA, USA), and dissolved in a binding buffer (50 mM NH_4_HCO_3_, 150 mM NaCl, 1 mM CaCl_2_, 1 mM MnCl_2_, pH 7.4). The mixture was rinsed with 1 × PBS, and peptides were released by boiling for 10 min. Two‐dimensional liquid chromatography/mass spectrometry (LC‐MS) was performed using LTQ Orbitrap MS (Thermo Fisher, San Jose, CA, USA). Data analysis was performed using the Byonic software program (Protein Metrics; San Carlos, CA, USA) and the MaxQuant software program as described previously [26].

### 2.8 Cytokine array analysis

KG1a, KG1a-TWIST1 or KG1a-ko-TWIST1 cells (2 × 10^5^) were cultured in 6 cm dishes for 24 h. The cells were then incubated in serum-free medium for an additional 24 h, and the supernatants were collected. The collected supernatants were centrifuged and 500 μL was subjected to the Proteome Profiler Human XL Cytokine Array kit (R&D Systems). The cytokine array was imaged using a luminescence imaging system (Tanon 4600, Tanon, Shanghai, China), and the signal intensity of the cytokines was normalized to the intensity of the positive controls.

### 2.9 Isolation of human plasma samples and analysis of IFN-γ levels

Human plasma was isolated from the BM blood of HD or MDS/AML patients. Blood samples were collected into precoated EDTA tubes and immediately centrifuged at room temperature for 15 min at 2000 g, and plasma samples were collected and frozen at −80 °C until further use. IFN-γ levels in resulting plasma were measured in triplicate using a human IFN-γ ELISA kit (Beyotime, Haimen, China). The intensity of the chromogenic reaction was determined at 490 nm using a plate reader (DeTie HBS-1096A, Nanjing, China).

### 2.10 MitoSox™ red mitochondrial superoxide indicator

A total of 5 × 10^5^ cells in suspension were incubated with MitoSOX™ (Invitrogen, CA, USA) for 10 min at 37 °C while being protected from light. Cells were then washed, stained with a final concentration of 2.5 μg/mL 4′,6-diamidino-2-phenylindole (Invitrogen) for 10 min while being protected from light. Finally, the cells were analyzed by FACS. prior to analyzed by FACS.

### 2.11 Intracellular ROS assessment

Intracellular ROS production was analyzed using a dichlorodihydrofluorescein diacetate (DCFH-DA) staining kit (Beyotime). Cells were treated with DCFH-DA solution for 30 min at 37 °C in the dark. ROS production was analyzed by FACS.

### 2.12 Determination of the mitochondrial membrane potential (ΔΨm)

The alteration of the ΔΨm in BMMSCs was analyzed using a JC-1 staining assay kit according to the manufacturer's instructions (Beyotime). Briefly, BMMSCs were collected, rinsed with PBS and stained with JC-1 (20 μg/ml) for 30 min at 37 °C in the dark. Cells were rinsed with staining buffer twice and subjected to FACS.

### 2.13 Statistical analysis

The Prism 5.0 statistical software program (GraphPad Software; La Jolla, CA, USA) was used for statistical analysis. Intergroup means were compared using Student's *t*-test, and differences at p < 0.05 were considered statistically significant. Each experiment was performed in triplicate. Data are presented as the mean ± SEM.

## 3 Results

### 3.1 Alteration of the osteogenic and adipogenic differentiation potential of BMMSCs in AML/MDS

**Fig. 1:**
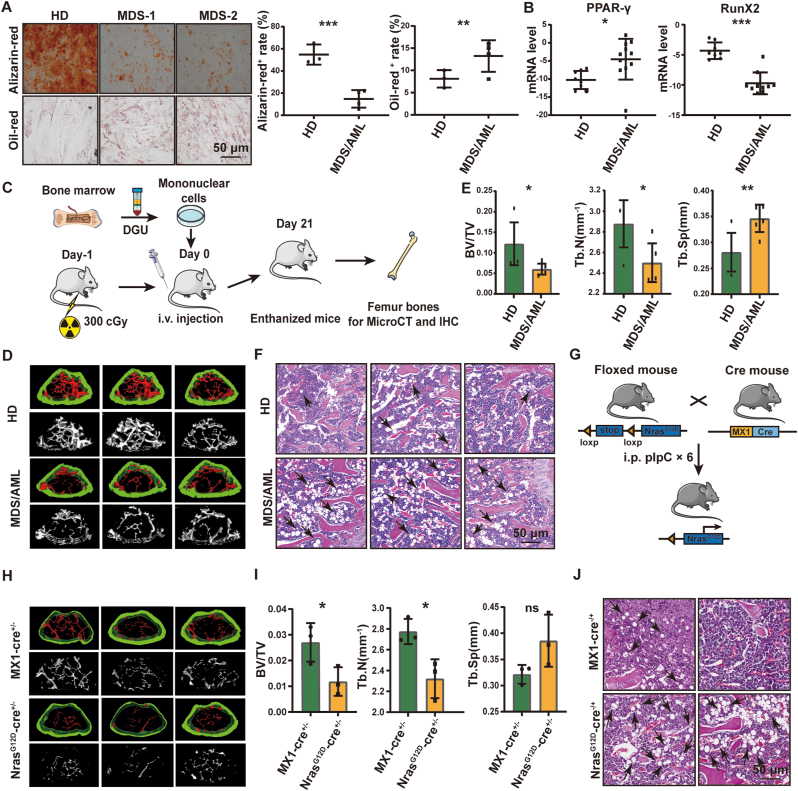
Alteration of the osteogenic and adipogenic differentiation potential of BMMSCs in AML/MDS **(A)** Primary BMMSCs derived from HD (n = 3) or MDS/AML patients (n = 4) were incubated with adipogenic or osteogenic differentiation medium. Lipid droplets were stained with oil red. Calcium nodules were stained with alizarin red. **(B)** The mRNA levels of PPAR-γ and RunX2 in mononuclear cells from HD (n = 8) and MDS/AML patients (n = 11). **(C)** Schematic diagram of the process for establishing the PDX mouse model. **(D)** Mouse femurs were observed by micro-CT. **(E)** The bone density of the femur was analyzed, and presented as the parameters bone volume/total volume (BV/TV), trabecular number (Tb.N) and trabecular separation (Tb.Sp). **(F)** HE staining of femurs injected with mononuclear cells from HDs or MDS/AML patients. Scale bar, 50 μm. **(G)** Schematic diagram of the process for establishing the *NrasG12D* transgenic mouse model. **(H)** Mouse femurs derived from MX1-cre^+/−^ and *Nras*^*G12D*^-cre^+/−^. **(I)** The BV/TV, Tb.N and Tb.Sp of femurs derived from MX1-cre^+/−^ and *Nras*^*G12D*^-cre^+/−^. **(J)** HE staining of femurs derived from MX1-cre^+/−^ and *Nras*^*G12D*^-cre^+/−^. Scale bar, 50 μm. (For interpretation of the references to colour in this figure legend, the reader is referred to the Web version of this article.)

The osteogenic differentiation of BMMSCs (CD45^−^, CD146^+^, CD105^+^, CD90^+^, CD44^+^) from MDS/AML patients was decreased significantly; in contrast, their adipogenic differentiation was increased (Fig. 1A, S1A). Consistently, the expression of the adipogenic marker PPAR-γ was upregulated, while that of the osteogenic marker RunX2 was downregulated (Fig. 1B). The femurs of mice injected with mononuclear cells from MDS/AML patients presented significant loss of bone trabeculae (Fig. 1C&D), lower bone volume fraction (BV/TV) and number of bone trabecular (Tb.N), and greater trabecular separation (Tb.Sp) (Fig. 1E). HE staining showed higher abundance of adipocyte in the BM of mice injected with MDS/AML mononuclear cells (Fig. 1F). Immunohistochemical analysis showed decreased expression of RunX2 and increased expression of PPAR-γ in the BM of MDS/AML mononuclear cells injected mice (Fig. S1B).

The *Nras*^*G12D*^*-cre*^*+/−*^ mice intercrossed from *LSL-Nras*^*G12D*^ and *Mx1-Cre* mice can exhibit MDS phenotype characterized by increased white blood counts, decreased hemoglobin (HGB) and enlarged spleen [27] (Fig. 1G, S1C-E). The spontaneous osteoporosis was observed in BM of these *Nras*^*G12D*^*-cre*^*+/−*^ mice (Fig. 1H), with lower trabecular BV/TV and Tb.N, and higher Tb.Sp, compared to WT controls (Fig. 1I). In the BM of *Nras*^*G12D*^*-cre*^*+/−*^ mice, fat accumulation (Fig. 1J), and increased PPAR-γ expression and decreased RunX2 expression (Fig. S1F), were also observed. The above results suggested an imbalance of osteogenesis and adipogenesis in BMMSCs from MDS/AML.

### 3.2 Shifted differentiation of BMMSC promoted MDS expansion

**Fig. 2:**
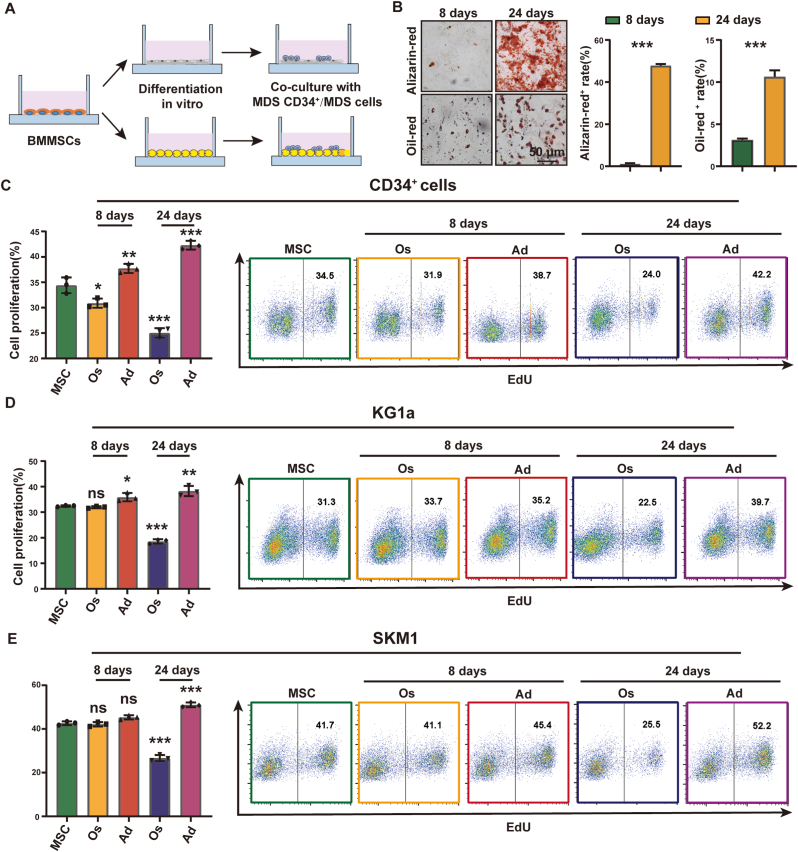
Differentiation shift of BMMSC differentiation promoted MDS expansion. **(A)** Co-cultured model. **(B)** Quantization of BMMSCs induced to adipogenic or osteogenic differentiation for 8 days and 24 days. Lipid droplets were stained with oil red. Calcium nodules were stained with alizarin red. **(C**–**E)** The proliferation of MDS cells (CD34^+^ cells derived from MDS patients, KG1a and SKM1) cocultured with adipocytes (Ad) or osteoblasts (Os) for 48 h was analyzed by flow cytometry. (For interpretation of the references to colour in this figure legend, the reader is referred to the Web version of this article.)

To investigate the effect of BMMSC differentiation defects on the proliferation of MDS cells, we co-cultured CD34^+^ cells from MDS patients with differentiated BMMSCs (Fig. 2A&B). Co-culture with adipogenic BMMSCs promoted the proliferation of CD34^+^ cells, while co-culture with osteogenic BMMSCs inhibited their proliferation (Fig. 2C). A similar phenomenon was observed in KG1a and SKM1 cell lines when co-cultured with differentiated BMMSCs (Fig. 2D–E).

### 3.3 Effect of TWIST1 in clonal cells on BMMSC differentiation

**Fig. 3:**
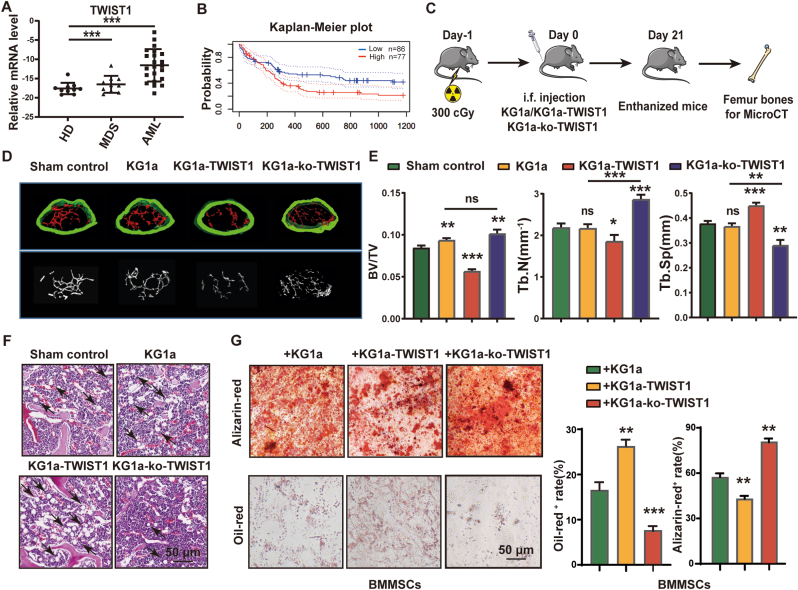
Effect of TWIST1 in malignant clonal cells on BMMSC differentiation (A) TWIST1 expression at the mRNA level in mononuclear cells from HDs (n = 10), MDS patients (n = 10) and AML patients (n = 21). (B) Kaplan–Meier overall survival curve to evaluate the prognostic significance of TWIST1 in the PrognoScan database. (C) Schematic of the process for establishing the xenotransplantation mouse model. (E) The bone density of the femur was presented ass BV/TV, Tb.N and Tb.Sp. (F) HE staining of femurs from KG1a- or KG1a-TWIST1-injected mice. (G) After co-culture with KG1a, KG1a-TWIST1 or KG1a-ko-TWIST1 cells for 48 h, BMMSCs were sorted and incubated with adipogenic or osteogenic differentiation medium. Lipid droplets were stained with oil red, and calcium nodules were stained with alizarin red. (For interpretation of the references to colour in this figure legend, the reader is referred to the Web version of this article.)

Consistent with our previous study [19], the expression of the transcription factor TWIST1 was increased in MDS, and exacerbated in AML (Fig. 3A). Higher expression of TWIST1 was related to poor prognosis in AML (Fig. 3B). Compared with KG1a cells, the injection of TWIST1-overexpressing KG1a cells (termed KG1a-TWIST1) resulted in bone loss, while injection of TWIST1-knock out KG1a cells (termed KG1a-ko-TWIST1) resulted in bone abundance in vivo (Fig. 3C–F). Lower expression of RunX2 and higher expression of PPAR-γ were observed in the femurs of mice injected with KG1a-TWIST1 (Fig. S2A).

Co-culture with KG1a-TWIST1 cells inhibited the osteogenic differentiation but promoted the adipogenic differentiation of BMMSCs *in vitro* (Fig. 3G). In contrast, co-culture with KG1a-ko-TWIST1 cells promoted the osteogenic differentiation but inhibited the adipogenic differentiation of BMMSCs (Fig. 3G). The expression of osteogenic markers (*RunX2*, *ALP* and *OCN*) were decreased in BMMSCs after co-cultured with KG1a-TWIST1 but increased in BMMSCs co-cultured with KG1a-ko-TWIST1 (Fig. S2C). We also found another MDS cell line SKM1 which knockdown TWIST1 (SKM1-shTWIST1) promoted the osteogenic differentiation but inhibited the adipogenic differentiation of BMMSCs (Figs. S2B and S2D). These results demonstrated that the elevated expression of TWIST1 in MDS/AML cells could determine the osteogenic/adipogenic differentiation of BMMSCs.

### 3.4 Abnormal oxidative phosphorylation in co-cultured BMMSCs

**Fig. 4:**
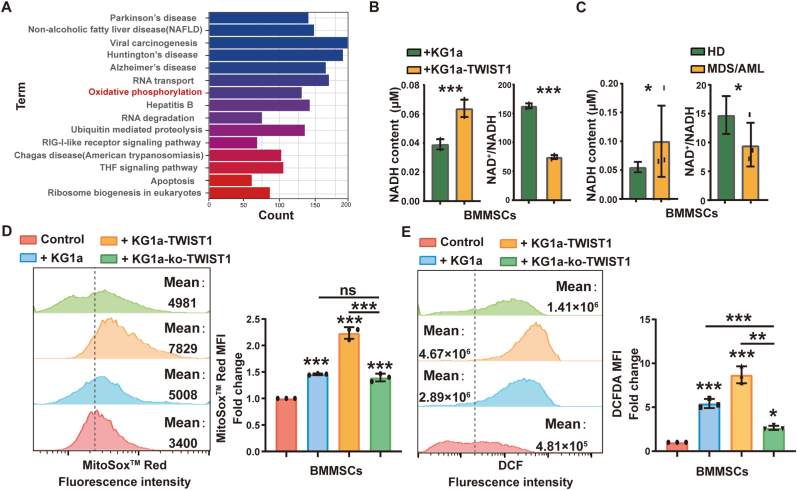
NADH alterations in MSCs induced by IFN-γ secretion in KG1a-TWIST1 cells **(A)**Top 15 enriched GO terms for 465 significantly differentially expressed genes in BMMSCs. **(B&C)** NADH and ratio of NAD^+^/NADH in BMMSCs after co-culture with KG1a or KG1a-TWIST1 cells (B) or mononuclear cells derived from HD (n = 3) and MDS/AML patients (n = 3) for 48 h (C). **(D&E)** Mitochondrial (D) and total (E) ROS levels were measured by flow cytometry. MitoSox™ and DCFDA geometric mean fluorescence intensity (MFI) and representative histograms of BMMSCs co-cultured with KG1a, KG1a-TWIST1 or KG1a-ko-TWIST1 for 48 h. BMMSCs alone was used as a control.

Using proteomics analysis, we were able to enrich differentially expressed proteins during the progression of oxidative phosphorylation (OXPHOS) in BMMSCs after co-culture with KG1a-TWIST1 (Fig. 4A, S3A-C). It is known that OXPHOS disruption is accompanied by a reduction in the NAD^+^/NADH ratio [28]. Interestingly, we observed a higher NADH level and a lower NAD^+^/NADH ratio in BMMSCs co-cultured with KG1a-TWIST1 or mononuclear cells derived from MDS/AML patients, compared to those co-cultured with KG1a or mononuclear cells derived from HD (Fig. 4B&C). These results suggested that co-culture with KG1a-TWIST1 resulted in disturbing NADH level in BMMSCs. NADH is a key component in cellular antioxidation system and NADH-dependent reactive oxygen species (ROS) generation from mitochondria is one of the critical mechanisms of ROS generation [29,30]. Therefore, mitochondrial superoxide anion production and total ROS levels were increased in BMMSCs co-cultured with KG1a-TWIST1, while they were decreased in BMMSCs co-cultured with KG1a-ko-TWIST1 (Fig. 4D&E). The same phenomenon was found in BMMSCs co-cultured with SKM1 and SKM1-shTWIST1 (Figs. S3D–E). These data suggested TWIST1 overexpressing MDS/AML cells may educate BMMSC differentiation by oxidative phosphorylation-dependent metabolic manner.

### 3.5 Elevated IFN-γ induced by TWIST1 increased ROS level in BMMSCs

**Fig. 5:**
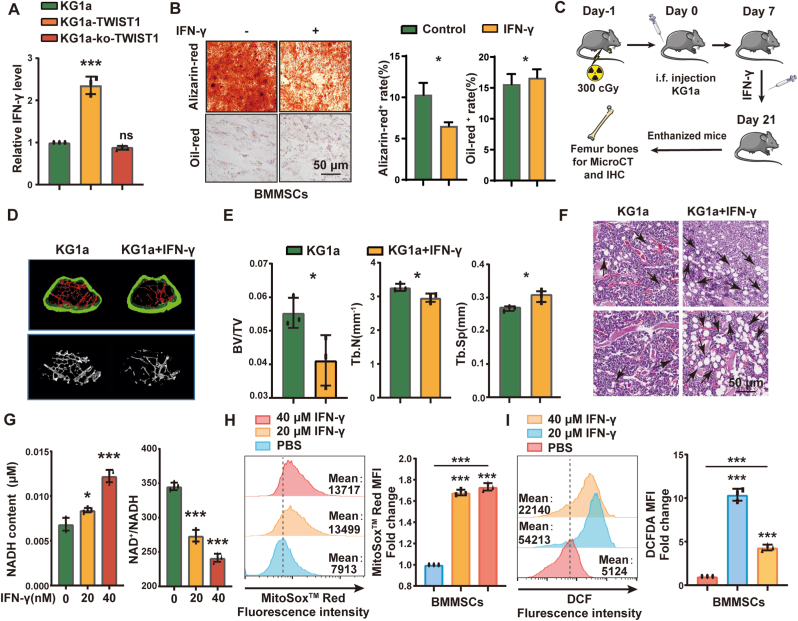
Secretion of IFN-γ is affected by TWIST1 **(A)** Elisa analysis of IFN-γ level in medium of KG1a, KG1a-TWIST1 and KG1a-ko-TWIST1 cells. **(B)** BMMSCs treated with IFN-γ were incubated with adipogenic or osteogenic differentiation medium. Lipid droplets were stained with oil red. Calcium nodules were stained with alizarin red. Scale bar, 50 μm. **(C)** Schematic of the xenotransplantation mouse model. Adult C57BL/6 mice were irradiated with 3 Gy and injected with 2 × 10^6^ KG1a cells and IFN-γ (2 mg/kg) by intrafemoral injection 3 times a week. The mice were sacrificed on day 21, and the femur was evaluated by micro-CT. **(D)** Mouse femurs were observed by micro-CT. **(E)** The bone density of femur bones from mice injected with KG1a cells and IFN-γ. **(F)** HE staining of femurs from KG1a cells and IFN-γ-injected mice. **(G)** NADH and ratio of NAD+/NADH in BMMSCs treated with 20 nM IFN-γ for 48 h **(H&I)** Mitochondrial (G) and total (H) ROS levels were measured by flow cytometry. (For interpretation of the references to colour in this figure legend, the reader is referred to the Web version of this article.)

As malignant cells can secrete cytokines that contribute to BMME remodeling, we found the increased secretion of IFN-γ level in medium of KG1a-TWIST1 compared to KG1a (Fig. 5A & Fig. S4D). The IFN-γ level in plasma of MDS/AML patients compared to HD, was significantly elevated (Fig. S4B). TCGA database also showed the upregulated expression of IFN-γ in AML (Fig. S4C). Combining with bioinformatics analysis, ChIP assay and luciferase assay, we found that TWIST1 could bind E-box 5 motifs of IFN-γ and activate its transcription (Fig. S4D&E).

When treated with IFN-γ, osteogenic differentiation of BMMSCs were significantly inhibited, while adipogenic differentiation was promoted (Fig. 5B). The irradiated mice injected with KG1a cells and IFN-γ (Fig. 5C) presented clearly inhibited osteogenic differentiation and more adipocyte abundance, as well as lower expression of RunX2 and higher expression of PPAR-γ (Fig. 5D–F, Fig. S4F). IFN-γ treatment also resulted in a higher NADH level and lower NAD+/NADH ratio (Fig. 5G), and increased mitochondrial superoxide anion production and total ROS level was increased in BMMSCs (Fig. 5H&I).

### 3.6 IFN-γ increase ROS level to mediate BMMSCs differentiation through STAT1 signaling

**Fig. 6:**
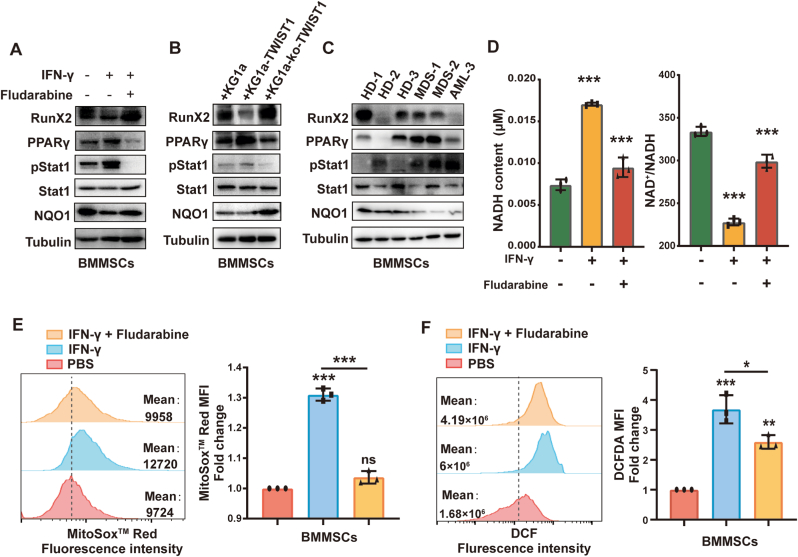
IFN-γ mediated the differentiation of MSCs through STAT1 signaling (A-C) Western blot analysis of RunX2, PPAR-γ, NQO1, Stat1 and p-Stat1 expression levels in BMMSCs (A) treated with 20 nM IFN-γ and 5 μM fludarabine or (B) co-cultured with KG1a, KG1a-TWIST1 or KG1a-ko-TWIST1 or (C) treated with plasma from HD and MDS/AML patients for 48 h. (D) NADH and ratio of NAD+/NADH in BMMSCs treated with IFN-γ for 48 h (E&F) Mitochondrial (E) and total (F) ROS levels were measured by flow cytometry.

IFN-γ can bind to IFN receptors and activates JAK1/JAK2/STAT1 signal transduction via phosphorylation of JAK and STAT1 [31]. As expected, p-STAT1(Tyr701) levels were significantly enhanced (Fig. 6A). The BM of irradiated mice injected with KG1a cells and IFN-γ also presented higher p-STAT1(Tyr701) level (Fig. S4G). Moreover, PPAR-γ expression was significantly increased, and RunX2 expression was clearly decreased in IFN-γ-treated BMMSCs (Fig. 6A). STAT1 signal pathway inhibitor (fludarabine) treatment reversed the abnormal expression of RunX2, PPAR-γ caused by IFN-γ. After co-cultured with KG1a-TWIST1 or treatment with MDS/AML plasma, BMMSCs showed decreased RunX2 levels, increased PPAR-γ levels, and activation of the STAT1 signaling pathway (Fig. 6B&C). We found that IFN-γ can stimulate ROS production compared to control (Fold change = 1.6) while total ROS increased about 10 times compared to control group (Fig. 5G&H). These results suggested alteration of ROS scavenging progress may serve as the dominant reason of increased ROS level in IFN-γ treated BMMSCs. STAT1 signaling pathway has been found to down-regulate quinone oxidoreductase 1 (NQO1), which function as ROS scavengers in breast cancer [31]. Here we also found that NQO1 was downregulated in IFN-γ or MDS/AML plasma treated or co-cultured BMMSCs (only with KG1a-TWIST1) and upregulated in BMMSCs treated with fludarabine or cocultured with KG1a-ko-TWIST1 (Fig. 6A–C). Fludarabine treatment reversed upregulated content of NADH, the decreased NAD^+^/NADH ratio and mitochondrial/total ROS level caused by IFN-γ (Fig. 6D–F). These data indicated IFN-γ could decrease NQO1 level to increase total ROS in BMMSCs through STAT1 signaling pathway.

### 3.7 Fludarabine and ROS scavenger reversed BMMSCs differentiation defects

**Fig. 7:**
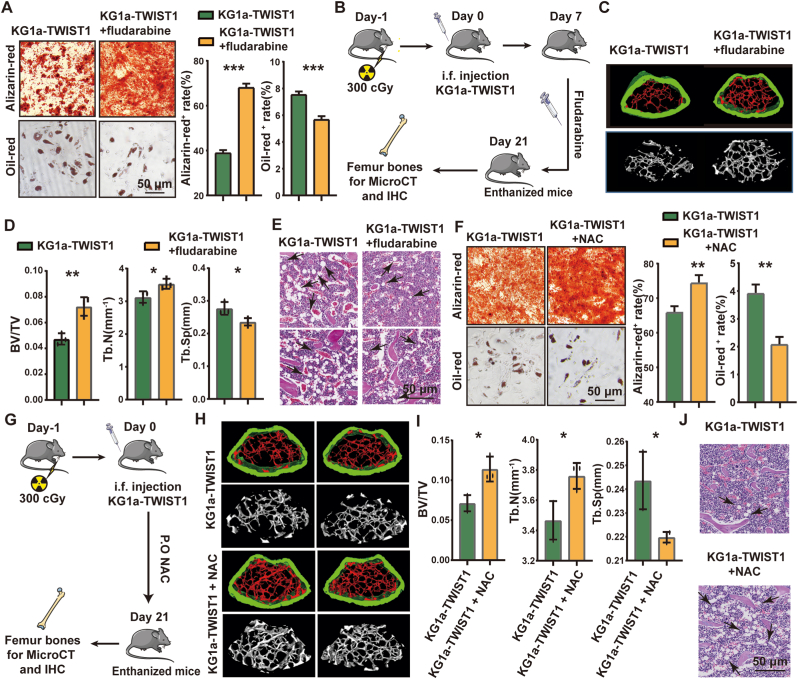
Stat1 signal pathway inhibitor and ROS scavenger reversed differentiation defects. (A) BMMSCs co-cultured KG1a-TWIST1 and then treated with Fludarabine were incubated with adipogenic or osteogenic differentiation medium. Scale bar, 50 μm. (B) Schematic of the xenotransplantation mouse model. Adult C57BL/6 mice were irradiated with 3 Gy and injected with 2 × 10^6^ KG1a-TWIST1 cells and fludarabine (1 mg/kg) by intrafemoral injection 3 times a week. The mice were sacrificed on day 21, and the femur was evaluated by micro-CT. (C) Mouse femurs were observed by micro-CT. (D)Analasis of the bone density of femur bones from mice injected with KG1a cells and Fludarabine.(E) HE staining of femurs from KG1a-TWIST1 cells and Fludarabine-injected mice. (F) BMMSCs treated with 10 mM NAC were incubated with adipogenic or osteogenic differentiation medium. Scale bar, 50 μm. (G) Schematic of the xenotransplantation mouse model. Adult C57BL/6 mice were irradiated with 3 Gy and injected with 2 × 10^6^ KG1a-TWIST1 cells and taken the water containing NAC (7 g/L) orally daily. The mice were sacrificed on day 21, and the femur was evaluated by micro-CT. (H) Mouse femurs were observed by micro-CT. (I) Analysis of the bone density of femur bones from mice treatedd with KG1a-TWIST1 and NAC. (J) HE staining of femurs from KG1a-TWIST1 cells and NAC -treated mice. Scale bar, 50 μm.

We next found fludarabine treated BMMSCs significantly stimulated osteoporosis but reduced adipogenesis (Fig. 7A), suggesting STAT1 signaling pathway have an impact on BMMSCs differentiation. Then, the irradiated mice were injected with KG1a-TWIST1 cells and treated with fludarabine (Fig. 7B). Osteogenic differentiation was clearly promoted, as indicated by increased trabecular BV/TV and Tb.N and decreased Tb.Sp values (Fig. 7C&D). HE staining showed the loss adipocyte in the BM of fludarabine treated mice (Fig. 7E). Meanwhile, the expression of RunX2 was increased and PPAR-γ was decreased in the BM of IFN-γ and Fludarabine-injected mice (Fig. S5A).

We then utilize N-Actyl-l-cysteine (NAC), one ROS scavenger, to mice injected with KG1a-TWIST1. NAC treatment significantly promoted osteogenesis and reduced adipogenesis in NAC treated BMMSCs (Fig. 7G–J). The KG1a-TWIST1 injected mice with NAC showed increased RunX2 expression and decreased PPAR-γ expression in the BM (Fig. S5B). These data suggested STAT1 signaling pathway inhibitor and ROS scavenger can reversed differentiation defects.

## 4 Discussion

BMMSCs, as a vital component of BMME, displayed abnormal differentiation capacities in terms of osteogenic or adipogenic differentiation in MDS/AML. The differentiation abnormality of osteopenia/osteoporosis has been observed in patients with MDS/AML [32,33]. Studies conducted using animal models have demonstrated that engrafted AML cells lead to increased mesenchymal stromal progenitor levels, impeding osteolineage development and bone formation [34]. Moreover, BMMSCs from MDS and AML patients have shown elevated adipogenic potential [35]. However, another research group found the leukemic cells-educated BMMSCs tend to differentiate into osteoblastic cells [9]. The inconsistent results are not surprising due to the complexity and heterogenicity of MDS/AML. Increasing evidence indicated that MDS/AML clonal cells induce various alterations in bone marrow niche and hijack the homeostasis of normal HSC to support leukemic progression [9,13,36,37]. For example, the accumulation of adipocytes in the educated bone marrow can further provide pro-tumoral support for AML blast proliferation [11]. Yet, the mechanisms of BMMSC differentiation abnormality in AML/MDS are still need to be clearly defined.

Dysfunctional crosstalk between BMMSCs and hematopoietic cells in the BMME can lead to abnormal hematopoiesis [38]. The BMME provides a number of soluble factors to support the survival and homing of hematopoietic cells, while malignant hematopoietic cells, such as MDS/AML clonal cells, can alter the BMME progressively to support their survival and proliferation. For instance, exosomes secreted by MDS or AML cells can transform the BMME into a leukemia-permissive BMME [34,39]. Our recent research has found MDS/AML patient-derived MSCs are phenotypically and functionally remodeled by myeloid cells and present a various glycosylation pattern, specifically a low bisecting GlcNAc modification, to modulate MCAM on stromal and affect proliferation of MDS/AML clonal cells [39]. In this study, we demonstrate that clonal MDS/AML cells hinder the differentiation of BMMSCs into osteoblasts but enhance their differentiation into adipocytes both *in vitro* and in vivo, suggesting the differentiation defects of BMMSCs are secondarily altered by the presence of MDS/AML clonal cells. We found that the oncogene TWIST1, highly expressed in AML and MDS [18,19], may drive the adipogenic differentiation of BMMSCs. TWIST1 is a basic helix-loop-helix (bHLH) transcription factor and plays essential and pivotal roles in both embryonic development and tumor initiation [40,41]. TWIST1 is a pivotal transcription factor that plays a central role in inducing epithelial-to-mesenchymal transition (EMT), a process closely associated with cell migration and invasion in cancer cells, ultimately promoting tumor progression [42]. Its phosphorylation is crucial for regulating its homo- and heterodimerization with other factors to control multiple cellular activities [43,44]. Our data suggested elevation of TWIST1 in MDS/AML clonal cells can contribute to increased secretion of IFN-γ. IFN-γ can act as a major mediator of antitumor immune responses, and it can affect the multipotential properties of MSCs [45,46]. Consistent with our findings, a high concentration of IFN-γ inhibits the osteogenic differentiation of MSCs *in vitro* [47,48]. In contrast, treatment with a neutralizing antibody against IFN-γ partially rescues BMMSC-mediated bone formation in C57BL/6 mice [47].

In our study, we showed MDS/AML clonal cells educate BMMSCs to use OXPHOS-related proteins during differentiation reprogramming. Mitochondrial OXPHOS is the main source of ROS, and deficiencies in the mitochondrial OXPHOS system can induce a variety of direct and secondary changes in metabolite homeostasis, such as increased ROS levels and decreased NAD+/NADH levels [49]. Previous study suggested a distinct link between ROS and BMMSC differentiation [50] and senescence [51]. Increased ROS levels were concluded to reduce the potential for osteogenic differentiation in MSCs derived from aged donors [52]. Therefore, observing respiratory enzyme complex activation and ROS in MDS/AML patients derived BMMSCs becomes logical.

IFN-γ secreted from MDS/AML cells can bind to IFN receptors, activating STAT1 signaling and downregulating the expression of NQO1 in BMMSCs [31]. Our data, together with previous results, confirm that IFN-γ promotes the generation of more ROS [53]. We also observed decreased NAD^+^/NADH ratios and reduced NQO1 expression accompanied by increased ROS levels in TWIST1 overexpressing clonal cells-educated BMMSCs. We believe that combination strategies that use essential ROS scavengers or inhibitor of STAT1 signaling pathway may be a potential way to eliminate the defects.

**Fig. 8:**
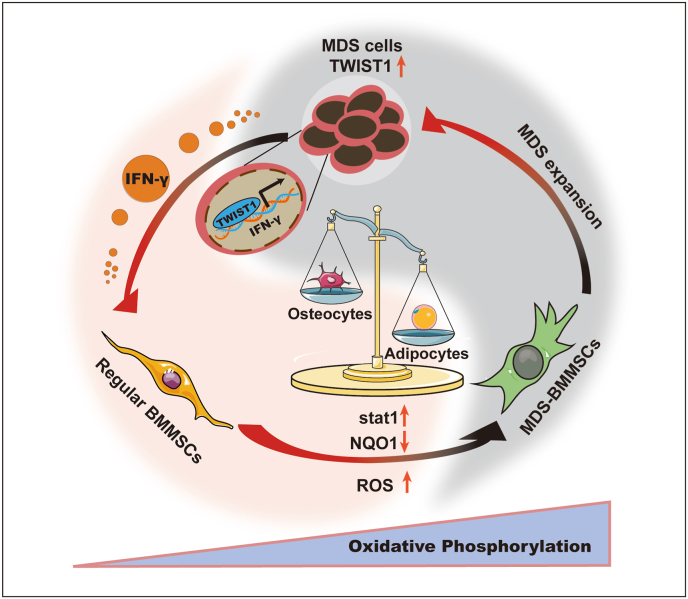
Schematic representation for reprogramming the differentiation of BMMSC by MDS/AML cells in the BMME.

Evolving evidence suggests that the BMMSCs function as a crucial factor in leukemogenesis, progression, and chemoresistance in a disease‐specific manner. Our study demonstrated that MDS/AML clonal cells with enhanced TWIST1 led to inhibition of osteolineage development and bone formation, while promoting adipogenic differentiation of BMMSCs through the secretion of IFN-γ. This imbalanced differentiation of BMMSCs created a pro-tumoral microenvironment to support the survival and growth of MDS/AML (Fig. 8). Therefore, targeting oncogenes such as TWIST1 in malignant clonal cells could potentially improve therapeutic strategies by remodeling the BMME in the treatment of MDS/AML and other hematopoietic malignancies.

## Author contributions

XL were responsible for conceiving and devising the study. HL and XL designed the experiments and analytical procedures. HL, XY, SF, KC and FY performed the experiments. YW provided the clinical samples. XL and FG supervised the research and collaborated on writing the manuscript. All authors read and approved the final manuscript.

## Declaration of competing interest

The authors declare that they have no known competing financial interests or personal relationships that could have appeared to influence the work reported in this paper.

## Data Availability

No data was used for the research described in the article.
