# Supplementary material for: Clonal MDS/AML cells with enhanced TWIST1 expression reprogram the differentiation of bone marrow MSCs
Source: Redox Biol. 2023 Sep 21;67:102900. doi: 10.1016/j.redox.2023.102900 (PMC10520935; doi:10.1016/j.redox.2023.102900)
Supplement: Multimedia component 1 [file mmc1.docx]

**Supplementary information**

**Clonal MDS/AML cells with enhanced TWIST1 expression reprogram the differentiation of bone marrow MSCs**

Hongjiao Li^1, #^, Yi Wang^2, #^, Xinwen Yu^1^, Shuang Feng^1^, Kaijing Chang^1^, Fenfang Yang^1^, Feng Guan^1^, Xiang Li^3*^

1. Key Laboratory of Resource Biology and Biotechnology of Western China, Ministry of Education; Provincial Key Laboratory of Biotechnology, College of Life Sciences, Northwest University, Xi'an, China.
2. Department of Hematology, Provincial People’s Hospital, Xi’an, China.
3. Institute of Hematology, School of Medicine, Northwest University, Xi’an, China.

# These authors contributed equally to this study.

The authors have declared that no conflict of interest exists.

Correspondence to Feng Guan (e-mail: guanfeng@nwu.edu.cn) or Xiang Li (e-mail: xiangli@nwu.edu.cn), Tel: +86-29-88303534, College of Life Science, Northwest University, 229 Taibai North Road, Xi’an, Shaanxi 710069, China

**Materials and methods**

Quantitative real-time PCR (qRT–PCR)

Total RNA was extracted using an RNA Pure Tissue & Cell Kit (Cwbiotech, Beijing, China), and cDNA was synthesized with a ReverTra Ace qPCR RT Kit (TOYOBO, Osaka, Japan). qRT-PCR was performed with Power SYBR Green Master Mix (Cwbiotech) on a Gentier 48R System (Tianlong Technology, Xi'an, China), using the primers provided in **Table S1**. The copy numbers of RunX2, ALP, OCN, LPL, and PPAR-γ were normalized to the expression of GAPDH.

SDS–PAGE and western blotting

Cells were lysed in radioimmunoprecipitation (RIPA) buffer supplemented with phenylmethanesulfonyl fluoride (PMSF). Equal amounts of protein (25 μg) were separated by SDS–PAGE and transferred onto polyvinylidene difluoride (PVDF) membranes (Bio-Rad; Hercules, CA, USA). After blocking with 3% bovine serum albumin (BSA, Sigma–Aldrich, St. Louis, MO, USA), the membranes were incubated overnight at 4°C in 5% nonfat milk/TBS-T containing primary antibodies against TWIST1 (Santa Cruz, CA, USA), RunX2, PPAR-γ Stat1, p-Stat1, NQO1 or Tubulin (Cell Signaling Technology, Beverly, MA), followed by the addition of a secondary antibody conjugated with horseradish peroxidase (HRP; Beyotime). Bands were visualized with a chemiluminescence kit and photographed using a bioluminescence imaging system (Tanon, Shanghai, China).

Chromatin immunoprecipitation assay (ChIP)

The ChIP assay was performed as described previously (1). In brief, KG1a-TWIST1 cells were cross-linked with 1% formaldehyde and the DNA was sheared to an average size of 200-500 bp using a sonicator. Immunoprecipitation was performed using antibodies against TWIST1 (Abcam, Cambridge, MA, USA) and IgG (Santa Cruz). Protein A/G agarose was added and incubated for 4 h. The samples were subsequently treated with proteinase K and RNase A (Beyotime, Haimen, China), followed by phenol/chloroform extraction and analysis via real-time PCR with the primers listed in **Table S1**.

**Table S1. Primer list**

| ChIP-E1-F | GCCTATCTGTCACCATCTC |
| --- | --- |
| ChIP-E1-R | AAGCTGATCAGGTCCAAAGG |
| ChIP-E2-F | GTCTCATCGTCAAAGGACC |
| ChIP-E2-R | CCTCCTCTGGCTGCTG |
| ChIP-E3-F | TACCAGGGCGAAGTGGG |
| ChIP-E3-R | TAGACTCCTTGGGTCCTTTG |
| ChIP-E4-F | GGCTGCCCCTTTGTAAAG |
| ChIP-E4-R | CACACCATTCAAGGACTGG |
| ChIP-E5-F | GGCAGTGCTGATCTAGAGC |
| ChIP-E5-R | CTAGGGCCTCTCAAACCT |
| ChIP-E6-F | CCACCATGCCTGGCT |
| ChIP-E6-R | GGCAGTGCTGATCTAGAGC |
| ChIP-E7-F | GACTAGTCATCCAATGTGCC |
| ChIP-E7-R | TGACTCAGTCTCCTTACCTG |
| RT-RunX2-F | TCTCCAACCCACGAATGCACTA |
| RT-RunX2-R | ATAGCGTGCTGCCATTCGAGGT |
| RT-LPL-F | CGCTCCATTCATCTCTTCA |
| RT-LPL-R | CTTGTTGATCTCATAGCCCA |
| RT-PPAR-γ-F | GGTTGAACAAGAGATGCCATTCT |
| RT-PPAR-γ-R | AATGCGAGTGGTCTTCCATCA |
| RT-ALP-F | TGGCTCTGCCTTTATTCCCTAGT |
| RT-ALP-R | AAATAAGGTGCTTTGGGAATCTGT |
| RT-OCN-F | GCCATCACCCTGTCTCCTAA |
| RT-OCN-R | GCTGTGGAGAAGACACACGA |
| RT-IFN-γ-F | TGGCTTTTCAGCTCTGCATCGT |
| RT-IFN-γ-R | TCCACACTCTTTTGGATGCTCTGGT |
| RT-MIP-1α-F | CAGCGAGTACCAGTCCCTTTT |
| RT-MIP-1α-R | CCTCGCTGCCTCCAAGA |
| RT-IL8-F | ACTCCAAACCTTTCCACCC |
| RT-IL8-R | AAACTTCTCCACAACCTCTG |
| RT-CCL1-F | GTTGCTTCTCATTTGCGGAG |
| RT-CCL1-R | GGTGTAGGGCTGGTAGTTTGC |
| IFN-γ-F | AATGTGGTAAAATCGATAAGTAGG  CCGGGTGCGG |
| IFN-γ-R | GCTCTCAAGGGCATCGGTCGAAGC  TGATCAGGTCCAAAGG |
| Mut-E1-F | ATTGTTCTGATACTCTGAAGATCAG |
| Mut-E1-R | CTGATCTTCAGAGTATCAGAACAAT |
| Mut-E2-F | CTACAACACCACAATGCCACAAA |
| Mut-E2-R | TTTGTGGCATTGTGGTGTTGTAG |
| Mut-E3-F | GAATGGCAACGGTGGGCATA |
| Mut-E3-R | TATGCCCACCGTTGCCATTC |
| Mut-E4-F | ACCACAAGACAATGATCAATGTG |
| Mut-E4-R | CACATTGATCATTGTCTTGTGGTT |
| Mut-E5-F | CGTTTTTACCTTGTTCCCAAC |
| Mut-E5-R | GTTGGGAACAAGGTAAAAACG |
| Mut-E6-F | TGGGATTAACGGTGTGAGCCAC |
| Mut-E6-R | GTGGCTCACACCGTTAATCCCA |
| Mut-E7-F | TTAATTTTACACTGAAGCATGGAAG |
| Mut-E7-R | CTTCCATGCTTCAGTGTAAAATTAA |

**Supplementary Figures**


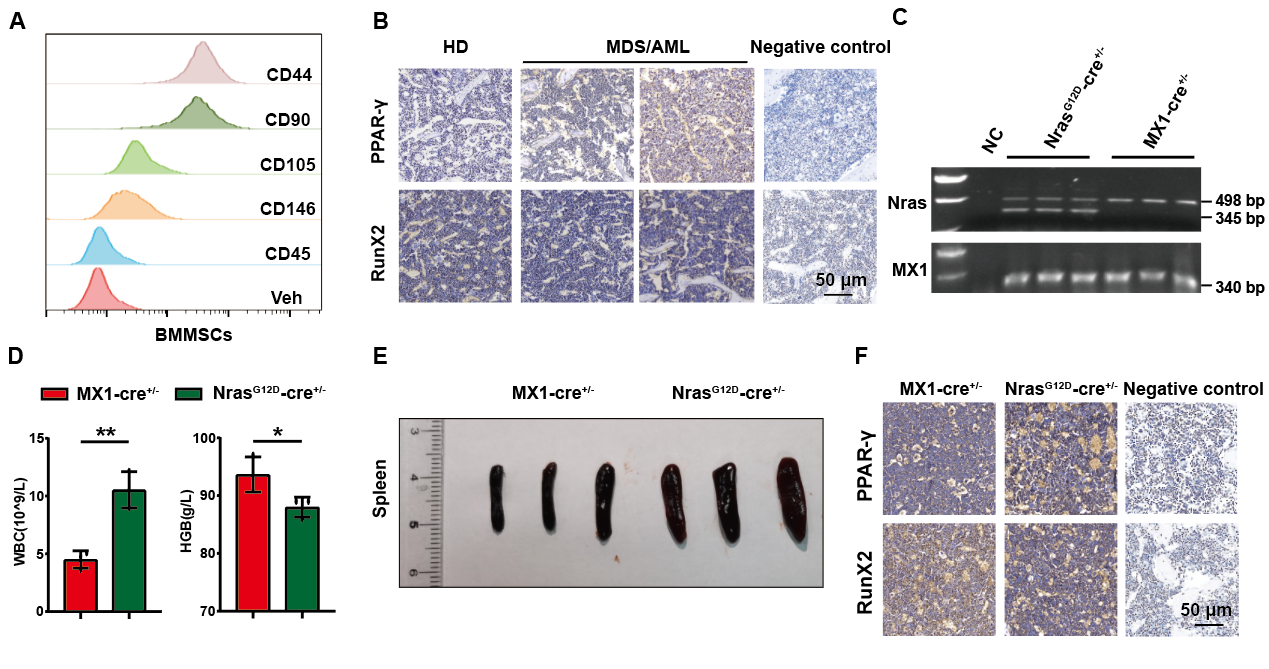


**Fig. S1 (A)** Characterization of BMMSCs from MDS patients by flow cytometry. **(B)** Immunohistochemical analysis of RunX2, PPAR-γ in femurs of WBCs from MDS/AML patients or HD injected mice. **(C)** The recombined Nras^G12D^-cre^+/-^ allele is detected in blood cells. The DNA fragments amplified Nras from the MX1-cre and Nras^G12D^-cre^+/-^ migrated at 493 bp and 345 bp respectively. **(D)** Peripheral blood analysis from MX1-cre and Nras^G12D^-cre^+/-^ mice **(E)** Spleen from from MX1-cre and Nras^G12D^-cre^+/-^ mice **(F)** Immunohistochemical analysis of RunX2, PPAR-γ in femurs from Nras^G12D^-cre^+/-^mice.


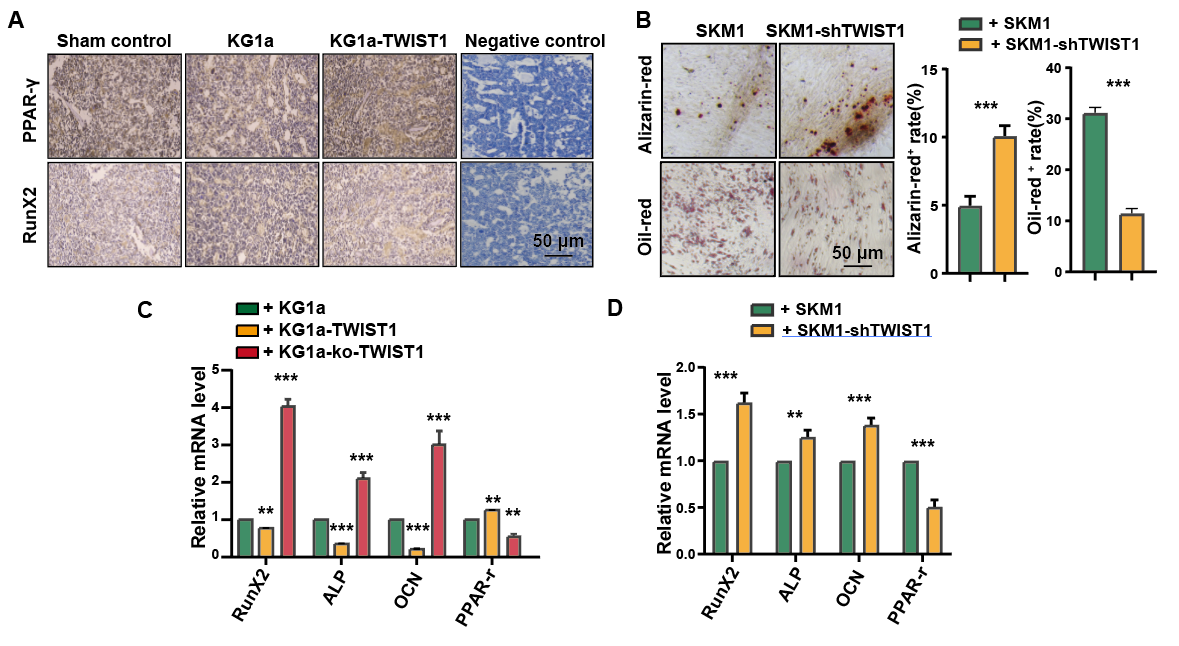


**Suppl. Fig. S2 (A)** Immunohistochemical analysis of RunX2 and PPAR-γ in the femurs of KG1a- or KG1a-TWIST1-injected mice. **(B)** After co-culture with SKM1 or SKM1-shTWIST1 cells for 48 h, BMMSCs were sorted and incubated with adipogenic or osteogenic differentiation medium. Lipid droplets were stained with oil red, and calcium nodules were stained with alizarin red. **(C)** The mRNA levels of RunX2, ALP, OCN and PPAR-γ in MSCs cocultured with KG1a, KG1a-TWIST1 or KG1a-ko-TWIST1 cells for 48 h. **(D)** The mRNA levels of RunX2, ALP, OCN and PPAR-γ in MSCs cocultured with SKM1 or SKM1-shTWIST1 cells for 48 h.


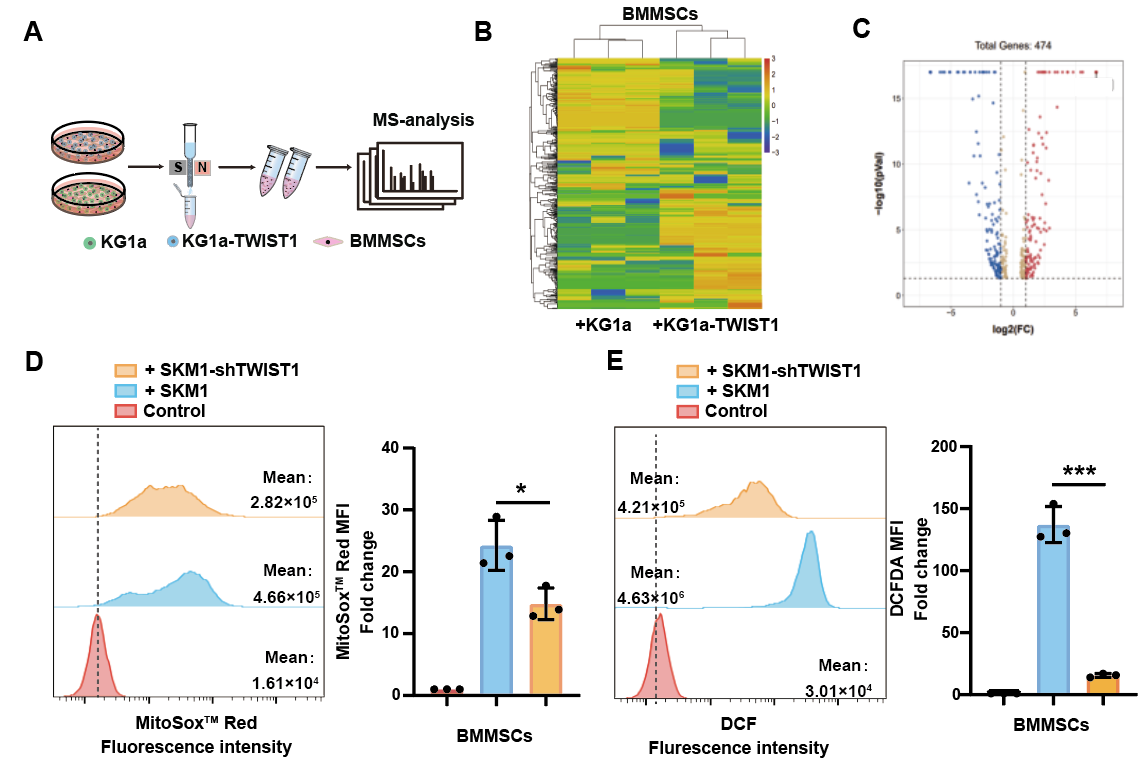


**Suppl. Fig. S3 (A)** BMMSCs were co-cultured with KG1a or KG1a-TWIST1 cells for 48 h and sorted by microbeads conjugated to a CD45 antibody, and differentially expressed proteins were analyzed by mass spectrometry. **(B)** Differentially expressed proteins in BMMSCs co-cultured with KG1a vs. KG1a-TWIST1 cells are shown as a heatmap. Red: high expression. Green: low expression. **(C)** Volcano plot of identified proteins. **(D&E)** Mitochondrial (D) and total (E) ROS levels were measured by flow cytometry. MitoSox^TM^ and DCFDA geometric mean fluorescence intensity (MFI) and representative histograms of BMMSCs co-cultured with SKM1 or SKM1-shTWIST1 for 48 h. BMMSCs alone was used as a control.


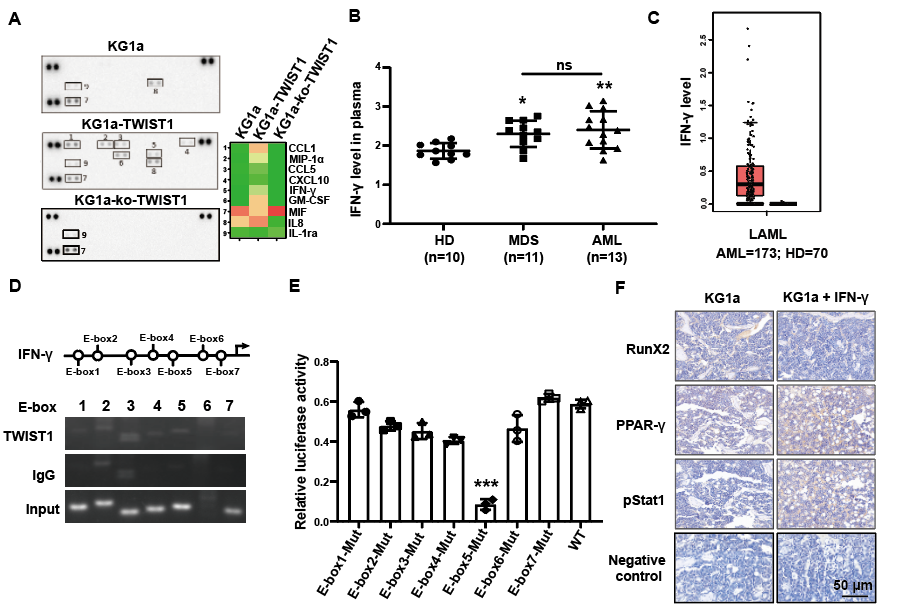


**Suppl. Fig. S4 (A)** The conditioned medium of KG1a, KG1a-TWIST1 and KG1a-ko-TWIST1 cells was analyzed by cytokine array. The differentially expressed cytokines are listed. **(B)** Elisa analysis of IFN-γ level in plasma of HD (n=10), MDS patients (n=11) and AML patients (n=13). **(C)** IFN-γ level in AML (n=173) and HD (n=70) analyzed by TCGA database. **(D)** The capacity of TWIST1 to bind to E-box motifs in the IFN-γ promotor was assayed by ChIP. The chromatin from KG1a-TWIST1 cells was digested and immunoprecipitated with a TWIST1 antibody. The specific DNA fragment was analyzed by PCR. Immunoprecipitation with IgG was used as a negative control. **(E)** HEK-293T cells were cotransfected with TWIST1 and two reporter plasmids; pGL3-basic (wild‐type or mutant IFN-γ promoter sequence) and pRL-TK. pRL-TK was used as a normal control. The luciferase activities of the transfected cells were assayed. **(F)** Immunohistochemical analysis of RunX2, PPAR-γ and pStat1 in the femurs of KG1a- or KG1a and IFN-γ -injected mice


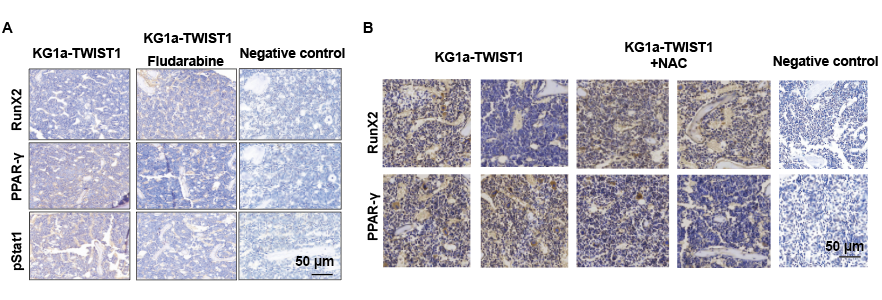


**Suppl. Fig. S5 (A)** Immunohistochemical analysis of RunX2, PPAR-γ and pStat1 in the femurs of KG1a-TWIST1- or KG1a-TWIST1 and Fludarabine -injected mice. **(B)** Immunohistochemical analysis of RunX2 and PPAR-γ in the femurs of KG1a-TWIST1- or KG1a-TWIST1 and NAC -treated mice.

**Reference**

1. Das PM, Ramachandran K, vanWert J, Singal R. Chromatin immunoprecipitation assay. Biotechniques. 2004 Dec;37(6):961-9.
